# Advanced Nanoscale Materials for Thermoelectric Applications

**DOI:** 10.3390/nano13243165

**Published:** 2023-12-18

**Authors:** Ting Zhang

**Affiliations:** 1 Nanjing Institute of Future Energy System, Nanjing 211135, China; zhangting@iet.cn; 2 Institute of Engineering Thermophysics, Chinese Academy of Sciences, Beijing 100190, China; 3 University of Chinese Academy of Sciences, Beijing 100049, China; 4 Innovation Academy for Light-Duty Gas Turbine, Chinese Academy of Sciences, Beijing 100190, China; 5 University of Chinese Academy of Sciences, Nanjing 211135, China

Recently, there has been growing academic interest in researching thermoelectric materials that exhibit energy conversion capability between thermal energy and electricity, providing solutions to energy crises and environmental pollution [1,2,3,4,5,6]. Generally, the efficiency of existing thermoelectric materials still has potential for improvement compared with traditional heat engines under the same operating conditions [7,8,9]. Nanomaterials, such as superlattices, quantum dots, nanowires, and nanocomposites, are considered one of the most effective materials for decoupling thermoelectric parameters, thus enhancing the performance of thermoelectric materials [10,11,12,13,14]. Although some relevant research has already been published, there is still great potential to further investigate the preparation, measurements, devices, and applications associated with thermoelectric nanoscale materials.

This Special Issue intends to summarize the advanced developments towards highly efficient thermoelectric nanomaterials and applications. In this Special Issue, we present nine high-quality original papers from the field of advanced nanoscale materials, with contributions from more than 50 authors worldwide.

In terms of inorganic thermoelectric materials, nanomaterials have an exceptional performance due to their narrow band gap, high electrical conductivity and low thermal conductivity. Zhao et al. found that a copper-based chalcogenide Cu_3_SbSe_4_ could achieve a maximum ZT value of 0.72 at 673 K due to a sulfur alloying effect, which widened the band gap, increased the effective carrier mass, and scattered phonons [Contribution 1]. Additionally, the use of nanowire networks is considered an effective method for manufacturing thermoelectric modules. Tristan et al. demonstrated a flexible thermoelectric module by embedding Co-Fe nanowires in a polymer film with a power factor of 4.7 mW/mK^2^ [Contribution 2]. The fabricated thermocouple operated as a Peltier cooler and achieved an equivalent cooling of 1.2 mW. Furthermore, thermoelectric fibers are promising for wearable applications due to their flexibility. Sun et al. successfully prepared flexible Cu-Se alloy core fibers using a thermal drawing method and obtained a high power factor of 1.2 mW/mK^2^, higher than that for bulk polycrystals [Contribution 3]. The Cu-Se fiber was applied to thermal–electric response with 5% measurement uncertainty. Using the same method, n-type Bi_2_Te_3_ fibers were prepared and the microstructure during the annealing process was explored [Contribution 4]. The reported Bi_2_Te_3_ fiber demonstrated an enhanced ZT value of 1.05 at room temperature after the Bridgman annealing processes.

Carbon nanotubes (CNTs) are widely used in the fields of electronics, energy and functional materials. The novel preparation technique makes it practical to develop high-thermoelectric-performance CNTs. Zimmerer et al. explored an environmentally friendly technique to coat single-walled carbon nanotubes (SWCNTs) with nickel using polydopamine (PDA) as an adhesion promoter [Contribution 5]. The results show that the SWCNTs modified by PDA have good dispersion and a homogeneous coating. The Seebeck coefficient of the obtained SWCNTs was reversed from positive to negative and reached −19 uV/K for the n-type application. Moreover, Almasoudi et al. polymerized CNTs with polypyrrole (PPy) in situ to form one-dimensional core–shell nanocomposites [Contribution 6]. The thermoelectric properties, including power factor and ZT value, were optimized to 0.36 mW/mK^2^ and 0.09, respectively. Using the prepared sample, a thermoelectric generator that can generate a maximum power of 24 nW at a temperature difference of 40 K was designed.

Hybrid thermoelectric materials combine the flexibility of organic materials with the high performance of inorganic materials and serve to further enhance the applications of flexible thermoelectric materials. For example, Li et al. produced boron nitride/polyetherimide (PEI) composite films via a casting–hot pressing method [Contribution 7]. The tensile strength of the composite film reached 102.7 MPa giving it a potential application in a flexible circuit substrate. Furthermore, Salah et al. developed a TiS_2_/organic hybrid superlattice (TOS), which had an optimized power factor of 0.1 mW/mK^2^ at a temperature of 233 K [Contribution 8]. Further studies suggest that TOS devices have better application prospects in cool environments than those at room temperature. In addition, Kim et al. prepared and studied several cellulose nanocrystal (CNC) aqueous solutions with surfactant aqueous solutions [Contribution 9]. As a result, the non-covalent dispersion method showed effective dispersion and stability.

In conclusion, this Special Issue, entitled “Advanced Nanoscale Materials for Thermoelectric Applications”, collates state-of-the-art achievements in the relevant fields of research, including CNTs, thermoelectric fibers and high-performance films. This Special Issue provides broad insights into the valuable research in these rapidly advancing and interdisciplinary fields.
